# Microfluidic multi-input reactor for biocatalytic synthesis using transketolase^☆^

**DOI:** 10.1016/j.molcatb.2013.05.016

**Published:** 2013-11

**Authors:** James Lawrence, Brian O'Sullivan, Gary J. Lye, Roland Wohlgemuth, Nicolas Szita

**Affiliations:** a Department of Biochemical Engineering, University College London, Torrington Place, London WC1E 7JE, UK; b Sigma–Aldrich, Industriestrasse 25, 9470 Buchs, Switzerland

**Keywords:** Micro reactor, Fed-batch, Transketolase, Laser fabrication, Microfluidic reactor

## Abstract

Biocatalytic synthesis in continuous-flow microreactors is of increasing interest for the production of specialty chemicals. However, the yield of production achievable in these reactors can be limited by the adverse effects of high substrate concentration on the biocatalyst, including inhibition and denaturation. Fed-batch reactors have been developed in order to overcome this problem, but no continuous-flow solution exists. We present the design of a novel multi-input microfluidic reactor, capable of substrate feeding at multiple points, as a first step towards overcoming these problems in a continuous-flow setting. Using the transketolase-(TK) catalysed reaction of lithium hydroxypyruvate (HPA) and glycolaldehyde (GA) to l-erythrulose (ERY), we demonstrate the transposition of a fed-batch substrate feeding strategy to our microfluidic reactor. We obtained a 4.5-fold increase in output concentration and a 5-fold increase in throughput compared with a single input reactor.

## Highlights

• Multi-point feeding strategy to overcome substrate inhibition in microreactors.
• Novel, flexible microreactor design offering numerous input configurations.
• Improved yield and throughput compared to previous T-channel reactor design.
• Use of rapid, flexible and widely available laser ablation fabrication technique.

## Graphical abstract

**Figure:**
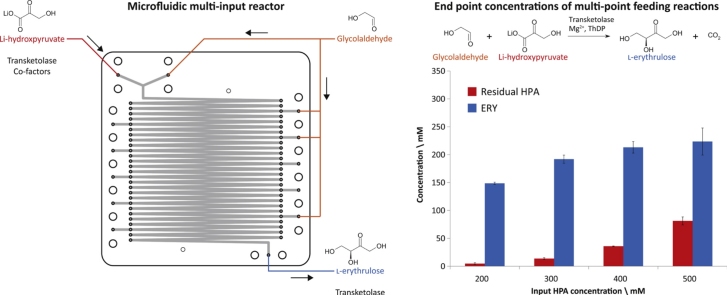

## 1 Introduction

Microfluidic reactors are of increased interest for preparative synthetic work due to a variety of advantages such as improved heat and mass transfer, mixing, safety, process intensification, scalability and reproducibility. As such they are being applied to a growing number of reactions in research and industry [1–4]. The use of microreactors for catalytic asymmetric synthesis has a high potential among the many different methodologies for the efficient and sustainable synthesis of valuable chiral compounds with improved control of reaction conditions and fast optimisation [5]. As biocatalytic asymmetric synthesis has proven to be a sustainable and viable methodology in organic synthesis and industrial manufacturing processes [6–9], efforts to combine the advantages of biocatalysis and micro-reactor technology in a mutually beneficial way have started to create numerous new application areas of interest, including resolution of racemic mixtures, reactions using hazardous substrates or two-phase systems [10–21].

Transketolases have been shown to be useful biocatalysts for creating new carbon–carbon bonds between aldehydes and the irreversible two-carbon donor lithium hydroxypyruvate (HPA) with high selectivity, broad substrate specificity and a high degree of conversion. They therefore represent a highly attractive and versatile biocatalytic platform technology with an ever-increasing range of applications [22–27]. Since the scale-up to viable manufacturing processes requires also work on the process design, reaction engineering and the optimisation of reaction conditions [28,29], the application of microreactor technology to transketolase-catalyzed carbon-carbon bond formation reactions is of fundamental interest [30].

In a previous publication, we have described the cascading of a microfluidic reactor and a filtration system for biocatalytic asymmetric synthesis using transketolase [31]. The system was able to fully convert HPA to the chiral product l-erythrulose (ERY) with high enantiomeric purity, as well as separating out the transketolase (TK) from the product, synthesising a pure pharmaceutical intermediate in a continuous-flow setting. However, the yield of ERY was low relative to the typical output that would be expected from chemical synthesis processes. Therefore it would be of great value to investigate improvements of yield and throughput in such devices.

The maximum product yield achievable with a T-channel reactor design, such as that used in our previous publication, is heavily dependent upon the effects of increased substrate concentration on the biocatalyst. These effects include inhibition and denaturation of the biocatalyst, with the latter causing clogging in continuous-flow reactors. In batch reactors, such effects are overcome by controlled feeding of the substrate solution at defined time points (fed-batch mode). Miniaturised fed-batch reactors with operation volumes of 12 mL [32] and 0.5 mL [33] exist, and could be employed to maintain the concentration of the substrate below inhibitory levels, allowing a higher product output. However these reactors cannot be operated in continuous-flow fashion, which limits their applicability for multi-step process integration. Additionally, the significant space-time-yield increases, characteristic of continuous-flow microreactors, cannot be exploited [34].

Reactor systems allowing injection of substrates at multiple points have been demonstrated for the purpose of controlling exothermic chemical reactions. These systems were designed for the continuous synthesis of allylcarbinol and of organometallic compounds, using multi-point feeding to control the formation of impurities and the generation of heat respectively [35–37]. However, such systems have not been applied to the problem of substrate inhibition in biocatalytic reactions.

Continuous-flow ‘loop-type’ microreactors designed to allow the recycling of unconverted substrates have previously been demonstrated [38,39]. It is possible to use these reactors to gradually feed substrate, however the continuous injection of substrate into the recycling loop at a single point means that a pure product stream is fundamentally difficult to achieve. The continuous removal of product also requires that the biocatalyst is either immobilised or removed by an in-line separation system, necessitating a more complex reactor design.

**Scheme 1:**
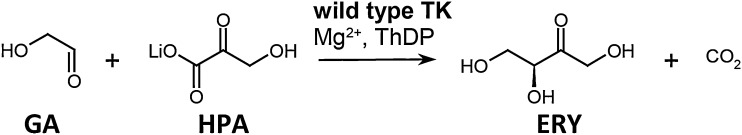
Reaction scheme. The transketolase-catalysed reaction of lithium hydroxypyruvate (HPA) and glycolaldehyde (GA) to l-erythrulose (ERY).

In this contribution, we combine a continuous-flow reactor and the principles of a fed-batch substrate feeding strategy for the first time at the microfluidic scale. We present the design of a novel microfluidic reactor capable of substrate feeding at multiple points. We demonstrate the application of the reactor to the TK-catalysed reaction of lithium hydroxypyruvate (HPA) and glycolaldehyde (GA) to l-erythrulose (ERY; Scheme 1).

## 2 Experimental

### 2.1 Reagents and analysis

Unless otherwise stated, all chemicals and reagents (Sigma–Aldrich, Gillingham, UK) were used without further purification.

Transketolase concentrations were measured by SDS-PAGE electrophoresis with 12% Tris–glycine resolving gel, using bovine serum albumin standards. 20 μg of total protein was applied to each lane and the samples were stained with Coomassie Blue R-250.

HPLC quantification of lithium hydroxypyruvate (HPA) and l-erythrulose (ERY) was performed on Aminex (Biorad, Hemel Hempstead, UK) ion-exchange column (HPX-87H, 300 mm × 7.8 mm), mobile phase: 0.1% (v/v) aqueous trifluoroacetic acid (TFA) at 0.6 mL min^−1^. HPA and ERY were detected by UV absorption at 210 nm.

### 2.2 Fabrication of the microfluidic multi-input reactor

The channels and cut-outs of the multi-input reactor (MIR) were fabricated in three layers of 1.5 mm poly(methylmethacrylate) (PMMA) using a CO_2_ laser marking head (Laserlines, Banbury, UK) with a maximum power of 25 W. The features were ablated with a power of 50% and a mark speed (laser tracking speed) of 200 mm s^−1^ and 10 mm s^−1^ for channels and cut-outs, respectively. The three layers were cleaned and thermally bonded (1.5 h, 105 °C, 90 min).

The interconnect blocks of the MIR were fabricated in 5 mm polycarbonate (PC) with a micromilling machine (Folken IND, Glendale, USA), using a 2 mm end mill (Kyocera, Kyoto, Japan) with a spindle speed of 10,000 rpm and feed rate of 80 mm min^−1^. M3 and M6 taps were used to prepare the interconnect blocks for use. Standard connection fittings were used to attach tubing (P-221, Upchurch Scientific, Oak Harbour, WA, USA).

Plugs used to seal unused auxiliary inputs were fabricated in poly(dimethylsiloxane) (PDMS; Sylgard 184, Dow Corning, Midland, USA). A mould was milled from 5 mm PMMA with a 2 mm tool, a spindle speed of 7000 rpm and feed rate of 40 mm min^−1^. The liquid polymer was prepared in a ratio of 10:1 (monomer to curing agent), cast, degassed and then cured at 90 °C for 2 h.

### 2.3 Preparation of transketolase lysate

Transketolase lysates were prepared according to the method of Matosevic et al. [40]. Overnight cultures of *E. coli* BL21gold (DE3) (with transketolase-producing plasmid pQR791) were grown in 2 L shake-flasks from inoculation of 400 mL Lysogeny Broth (LB) with 1 mL of concentrated cell suspension in LB-glycerol stock solution (25%, v/v, glycerol, stored at −80 °C until inoculation). This was incubated for 20–24 h at 37 °C, until the bacterial growth had reached stationary phase as confirmed by optical density measurements. The contents of the flask were transferred to 50 mL falcon tubes and centrifuged at 5000 rpm for 10 min. The supernatant was discarded and the cell paste was frozen at −80 °C until needed for lysis and purification.

For lysis, the cell paste was resuspended in 2 mL 50 mM Tris–HCl buffer, cooled on ice and sonicated (10 cycles of 10 s on, 10 s off) with a sonication probe (Soniprep 150, Sanyo, Japan). The suspension was then centrifuged at 5000 rpm for 10 min and the supernatant containing the enzyme was stored at −20 °C until required.

### 2.4 Continuous-flow microfluidic reaction of HPA to ERY (with multiple GA inputs)

**Scheme 2:**
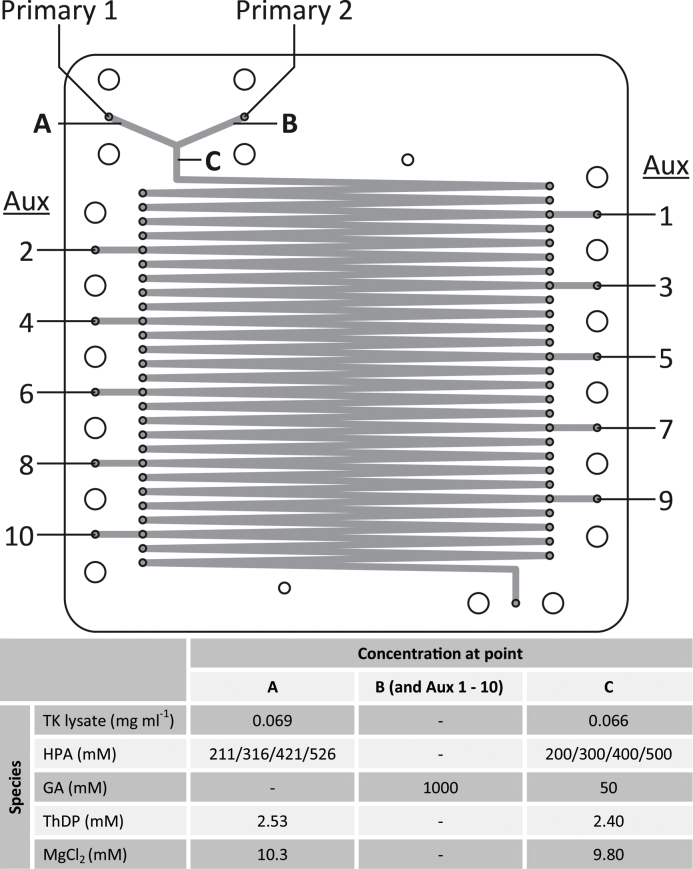
Reactor input layout and in-channel concentrations. Layout of reactor inputs referred to in Table 1 and concentrations found at inputs and in main reaction channel.

Two separate solutions were used to perform the reaction. The main reaction mixture (solution A) consisted of 0.069 mg mL^−1^ clarified transketolase lysate, different HPA concentrations (211/316/421/526 mM HPA, depending on experiment), 2.53 mM thiamine diphosphate (ThDP) and 10.3 mM MgCl_2_ in 50 mM Tris–HCl buffer, pH 7. The concentrations of the solutes were chosen such that they would be diluted to the following concentrations once combined with the first GA input: 0.066 mg mL^−1^ clarified transketolase lysate, 200/300/400/500 mM HPA, 2.4 mM thiamine diphosphate (TDP) and 9.8 mM MgCl_2_. The supplementary GA solution (solution B) consisted of 1 M GA in 50 mM Tris–HCl buffer, pH 7 (Scheme 2).

The MIR was primed with Tris–HCl buffer (50 mM, pH 7). Solution A was pumped into the first primary input of the reactor with a single-drive syringe pump. Solution B was pumped into the second primary input, along with a number of auxiliary inputs, using a dual-drive syringe pump adapted to fit ten 1 mL syringes. The flow rates and the number of auxiliary inputs used were dependent upon the desired residence time and input HPA concentration (Table 1).

The reactor was allowed to equilibrate for 2.5 residence times before sampling began. Samples were collected in pre-weighed vials containing 270 μL 0.1% (v/v) aqueous trifluoroacetic acid (TFA). The quenched samples were weighed, centrifuged and the supernatant was diluted 1:1 with 0.1% TFA before being analysed by HPLC as described in Section 2.1.

### 2.5 Fed-batch microplate reactions

Fed-batch reactions were performed in 96-well microplates. 200 μL of solution A was made up in each well. 10 μL of solution B was manually added to each well to start the reactions. Further 10 μL doses of solution B were then manually added to the reaction mixtures at specific time points, corresponding with the feeding strategy used in the continuous-flow reactions. Endpoint samples were taken after the reactions had run for the equivalent residence times, diluting 30 μL of reaction mixture in 270 μL 0.1% TFA. Samples were centrifuged before being analysed by HPLC.

### 2.6 12-h continuous production of ERY

The experiment was run at the conditions of the 500 mM HPA conversion (Table 1), using the same solutions and reactor setup as described in Section 2.5. The output tubing from the MIR was connected to a RotAXYS auto-sampling system (Cetoni GmbH, Korbussen, Germany), programmed to take 30 μL samples every hour from the start of the reaction. 12 wells of a 96-well plate were each filled with 270 μL of 0.1% TFA to quench the samples. Samples were centrifuged before being analysed by HPLC.

## 3 Results and discussion

### 3.1 Reactor design

For a typical microreactor consisting of a microchannel with two (or occasionally more) inputs at one end of the channel, the substrate concentration is defined at a single point (the input) and cannot be supplemented further. In such reactor designs, a higher concentration of product is obtained by increasing the concentration of substrate at the input. However, in biocatalysis, the effects of increased substrate concentrations, such as substrate inhibition and protein denaturation, set limits on the amount of substrate that can be added at a single point.

In the case of the TK-catalysed synthesis of ERY, we have observed an increase in reaction rate and yield with increased HPA and GA input, up to concentrations of 300 mM GA. It has previously been shown that GA concentrations of 500 mM GA cause a significant reduction in the activity half-life of TK, to 200 min as compared with 400 min for a concentration of 250 mM [41].

From our own observations when performing microfluidic reactions with GA concentrations of 500 mM, it becomes clear from visual inspection that a build-up of solid material is occurring, causing the reactor channel to clog. This severely impairs the functionality of the reactor, usually halting the flow before it has time to equilibrate. We therefore decided to explore whether feeding strategies, analogous to those used in fed-batch processes, could be used in a continuous-flow microreactor to overcome these limitations. It would thus be worthwhile investigating how to overcome these problems in order to remove the limitations on the yield of product that is achievable in biocatalytic microreactors.

**Fig. 1:**
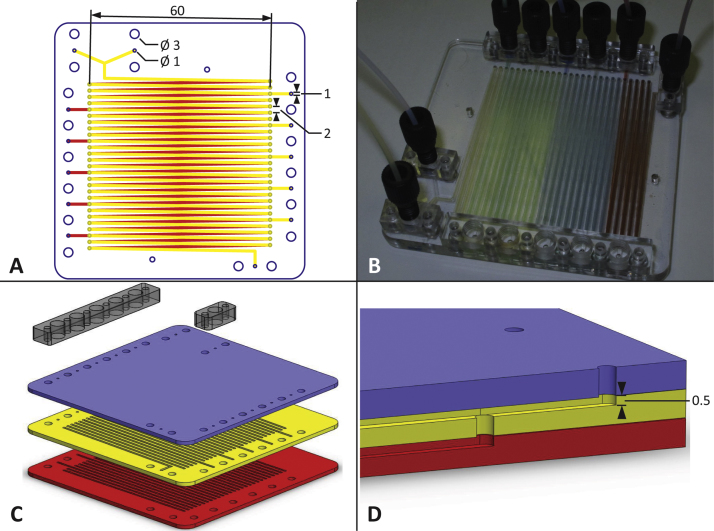
Design of the microfluidic multi-input reactor (MIR). (A) Top-down layout of channels in the reactor; shaded areas indicate ablated channels, lines indicate cuts through the substrate, dimensions in mm; (B) photo of the assembled MIR, showing interconnect bars with (back) and without (front) fittings attached; colored dyes were used to visualise the channels (dyes with different colors illustrate the several inputs of the MIR, but are not related to the color scheme of figures A, C, and D); (C) exploded view of reactor design also showing interconnect ports (fabricated in PC); (D) section view of the microfluidic reactor showing the ‘through-holes’ which connect the channels on different layers, dimensions in mm. (For interpretation of the references to color in text, the reader is referred to the web version of the article.)

To achieve this, we designed a microfluidic reactor with multiple ports, capable of feeding the total amount of substrate across multiple inputs, and through this enhance the flexibility of microreactors for biocatalytic synthesis. Fig. 1 illustrates the design of the reactor. The design consisted of one main channel with a total volume of 1650 μL fabricated in poly(methylmethacrylate) (PMMA), with two primary inputs at the front end of the channel where the biocatalyst and substrate were combined to start the reaction (Fig. 1A). Ten further auxiliary inputs were spaced evenly along the channel, where further substrate could be added. The inputs were designed for standard fittings, facilitating the easy reconfiguration of the reactor. PDMS plugs were also designed that were compatible with the standard inputs, allowing any unused inputs to be reliably and reversibly sealed (Fig. 1B). In this way the reactor could easily be configured to test a range of different feeding strategies.

To enable the conversion of large amounts of substrate, the reactor was designed to be operated with long residence times, and hence required a relatively large total volume. At the same time, to allow it to be fabricated on most available laser ablation systems, the outer dimensions of the reactor needed to be limited to 85 mm × 85 mm. We therefore opted for an elegant design which divided the channel over two layers, with ‘through-holes’ ablated into the middle layer to allow fluid to ascend and descend (Fig. 1C and D). All channels in the reactor had a cross section of 1 mm × 0.5 mm, and the length of individual sections of the main channel was 60 mm. Sections of channel on the middle (yellow in Fig. 1) layer were aligned 91° from the normal and channels on the base (red) layer were aligned 269° from normal. When bonded together, the channels on the different layers aligned to form a single channel.

Laser ablation was chosen as the fabrication method for its rapidity. Using the laser, it was possible to fabricate the comparatively complex middle layer in less than 6 min and to complete all three layers of the reactor in around 15 min. This fits well with the ‘rapid prototyping’ paradigm that is a common feature of microfluidics. A video showing the rapid laser fabrication of the middle layer can be found in the Electronic Supplementary Information 1. Given the number of channels and other features involved, fabricating the same design using a micro milling machine, another commonly used tool for rapid fabrication, would have taken a few days. As a further advantage, the low cost of PMMA makes the reactors very inexpensive to produce, meaning that they can easily be disposed of and replaced at the end of their useful life.

The increasing availability of laser ablation systems means that the reactor could be fabricated by groups without specialist microfluidics knowledge, and the modular design facilitates easy adaptation to suit the needs of different biocatalytic reactions. The offset of the auxiliary input ports from the main reactor channel provides an added benefit, allowing the structure of the main channel to be changed without modification of the port layout. Decoupling the inputs from the channel in this way means that the residence volume between individual ports can be adjusted, for example to allow for shorter residence times between inputs earlier in the reaction. Combined with the rapidity of the fabrication method, this would allow the production of reactors that were tailored to the exact substrate feeding schedule required by the reaction. Additionally, the reactor could be scaled up by simple modifications to the design and by use of a larger laser ablation system. This would then allow bench-top or even pilot scale production in continuous-flow, which is of interest to the chemical and biochemical industries [4].

The flow regime within the main reactor channel was determined to be laminar, with a Reynolds number of 2.2 × 10^−4^ at a flow rate of 10 μL min^−1^ (calculated using the viscosity and density of water). This was confirmed by confocal imaging of diffusion at a junction between an auxiliary input and the main channel, where the fluorescent tracer dye fed though the auxiliary was shown to form a lamina over the bulk fluid in the channel (see Electronic Supplementary Information 2, Fig. S2).

Despite the laminar regime and the absence of mixing structures, the tracer appeared to be evenly distributed throughout the channel within a residence time of 1 min, equivalent to less than half the length of a single channel section at the highest flow rates used in the synthesis experiments. This is in agreement with the calculation of the diffusional length of the tracer in the channel, which predicts that it will have propagated 0.39 mm across the channel depth in 1 min (where the diffusion coefficient of fluorescein in water is 0.64 × 10^−9^ m^2^ s^−1^ [42]).

### 3.2 Performance of the multi-input reactor

The conversion of four different input concentrations of HPA was carried out in the MIR and in 96-well microplates, using four different feeding strategies. In each strategy the configuration or timing of GA feeding points was altered in order to avoid inhibitory effects on the enzyme whilst aiming for complete conversion to ERY. The process of selecting GA feeding points is explained further in Electronic Supplementary Information 3.

The configurations used for each HPA input concentration are shown in Table 1. The flexibility of the reactor was such that each of these strategies could be tested with a minimal amount of effort in setting up, simply by changing the auxiliary connections and flow rates as required.

**Fig. 2:**
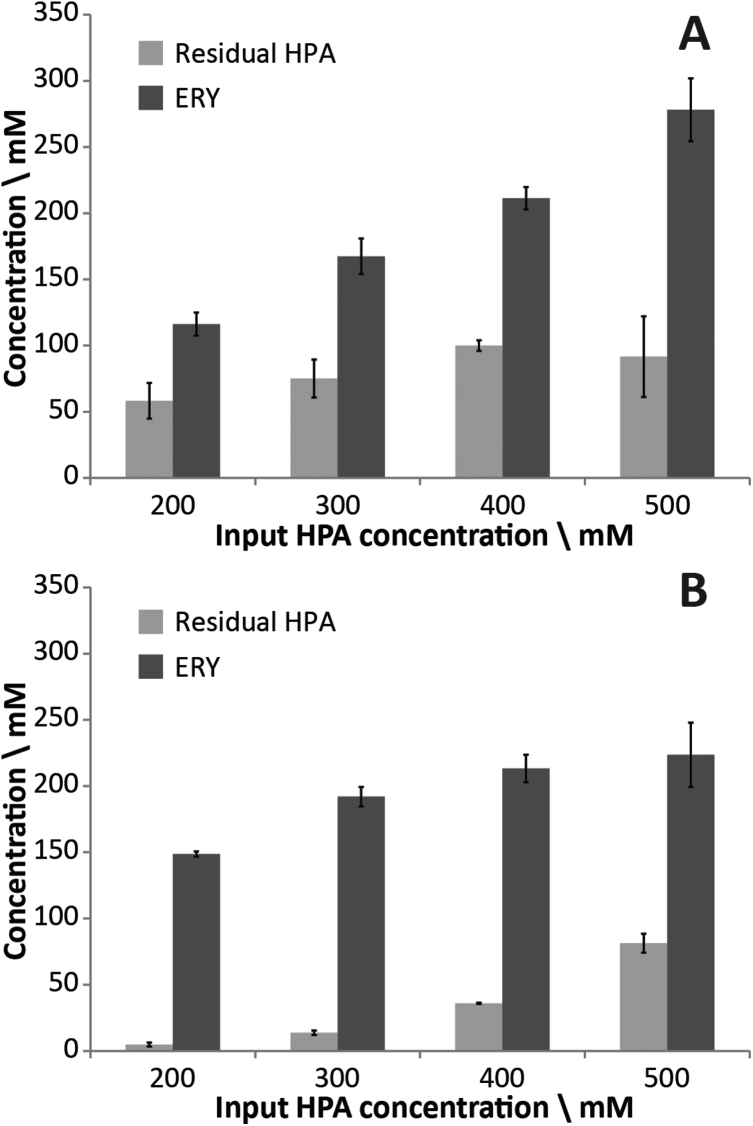
End point concentrations of HPA and ERY from TK-catalysed reactions. (A) Fed-batch 96-well plates; (B) microfluidic multi-input microfluidic reactor. Error bars are standard deviation (*N* = 3). The residence times used for the conversion of different input HPA concentrations are given in Table 1.

Fig. 2 shows the endpoint concentrations of HPA and ERY from the MIR and fed-batch microplate well reactions. Compared with our own single substrate input microfluidic reactor [31], the multi-point feeding strategy increased the yield of product from 50 mM to a maximum of 224 mM with the MIR (Fig. 2B). Compensating for the dilution caused by the nine GA inputs used in the feeding strategy, the maximum concentration of ERY generated is 313 mM.

**Fig. 3:**
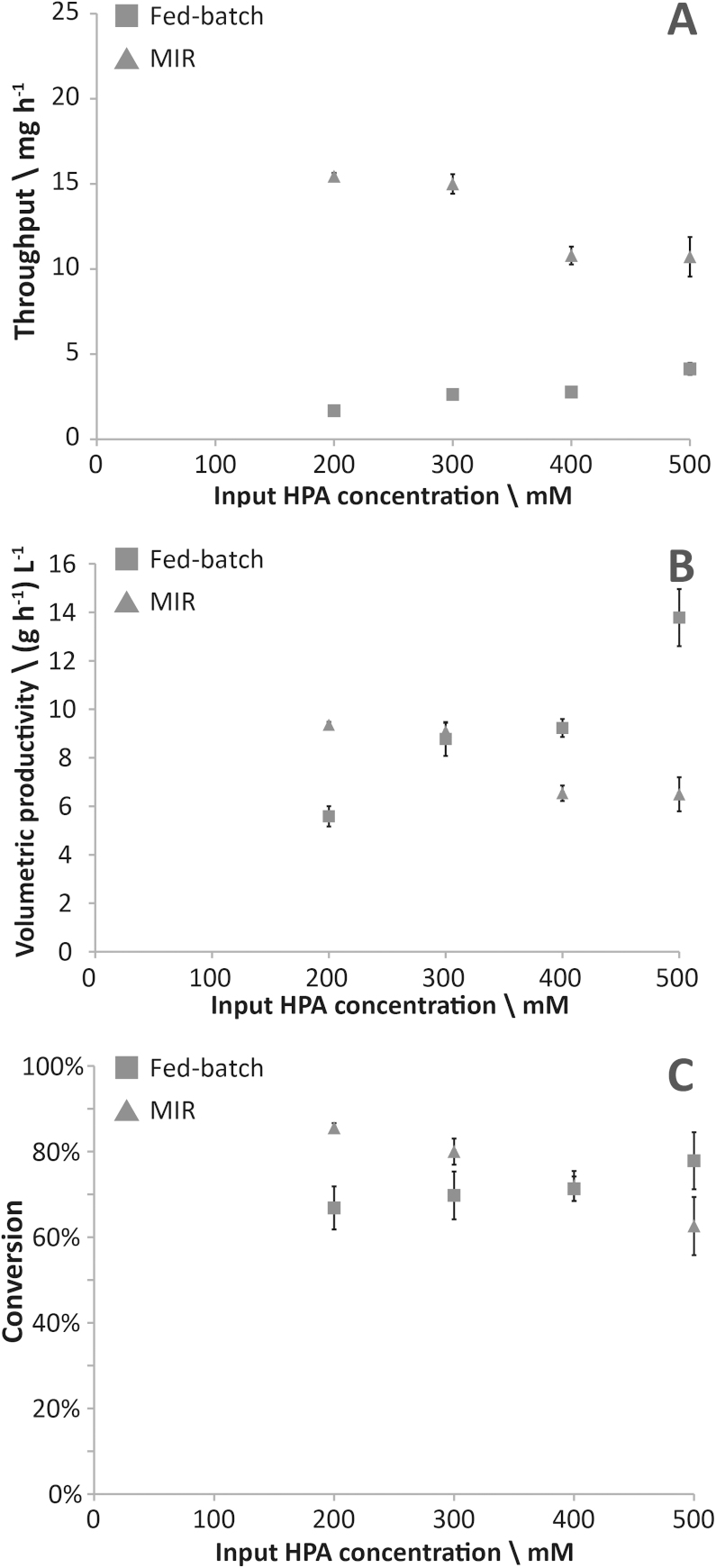
Throughput, volumetric productivity and conversion data for different reaction modes.(A) Throughput of ERY from each system; (B) volumetric productivity (C) percentage conversion of HPA to ERY. Squares (■) = fed-batch 96-well plates, triangles (▴) = microfluidic multi-input reactor. Error bars refer to standard deviation (*N* = 3).

Fig. 3 shows the conversion, throughput and volumetric productivity data from the MIR and equivalent fed-batch microplate well reactions. Comparing the output from the respective systems, we see a very large improvement in the throughput of ERY from the MIR, regardless of the input HPA concentration used, with the highest observed increase being 8-fold at the lowest concentration (Fig. 3A). By comparison with our single substrate input reactor we also see an increase in throughput from 3 mg h^−1^ to 15 mg h^−1^. This clearly shows the improvements in throughput and space-time yield that are the foremost advantage of continuous flow reactors over batch or fed-batch reactors, which is in agreement with similar comparisons from the literature [34]. A continuous flow system is also of great interest for use in multi-step synthesis processes, where a cascade of separate reactors can be created to perform a complex conversion. The MIR would be well-suited to this, having standard interconnect ports that would allow connection to other reactors or intermediate purification steps.

The volumetric productivity and conversion achieved in the MIR was higher than that of the fed-batch microplate at lower input HPA concentrations, reaching a maximum of 9.4 (g h^−1^) L^−1^ and 86% respectively (Fig. 3B and C). At higher HPA concentrations the conversion and productivity of the fed-batch microplate reactions surpassed that of the MIR. This may have been caused by decreasing pH as the reaction progressed, due to the evolution of CO_2_ as a by-product, which was released to the headspace in the microplates but remained in solution in the MIR.

A reaction system designed for synthesis should aim to reach 100% conversion in order to minimise the downstream requirement for purification, or unintended side reactions when used as part of a multi-step process. Any change in pH could be mitigated in a manner analogous to that of a pH-stat reactor. This could be accomplished by using some of the auxiliary inputs on the MIR to feed a base solution, thereby counteracting the pH drop and allowing complete conversion of substrate to product. However, to investigate this further would require integration of online pH monitoring into the MIR; a technology that has thus far only been used in cellular microreactors [43].

**Fig. 4:**
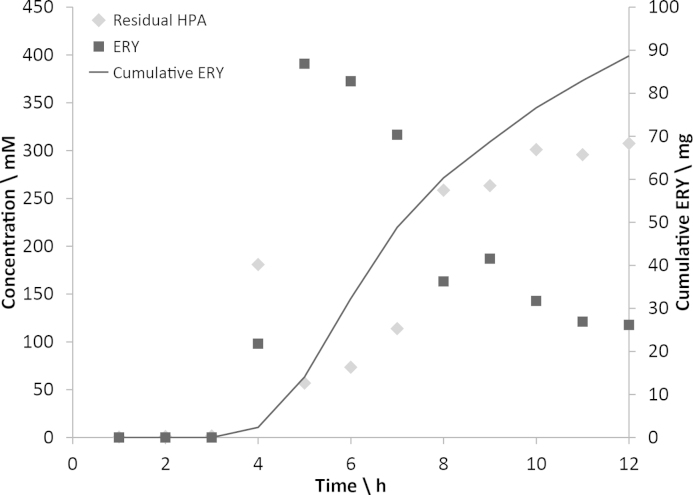
Profile of output concentrations from a 12-h continuous reaction run. Output concentrations of HPA (diamonds, ♦) and ERY (squares, ■) from reaction run for 12 h using 500 mM input HPA concentration, 9 GA inputs and 286-min residence time (*N* = 1). Time refers to the number of hours elapsed after flow was started. No HPA/ERY was recorded for the first 3 h as the reaction mixture had not yet reached the output. The line indicates an estimate of the cumulative weight of ERY produced over the course of the run.

Fig. 4 shows the output concentration profiles of HPA and ERY from a reaction performed in the MIR using the feeding strategy developed for a 500 mM HPA input concentration. The reaction was sampled every hour with an automated sampling arm. The reaction equilibrated between hour 4 and 5 (as might be expected given that the residence time was 286 min), with a maximum output concentration of around 400 mM ERY. An estimated 90 mg of ERY was produced over the course of the experiment. The output concentration drops continuously between hours 5 and 12, suggesting decay in TK activity due to exposure to room temperature conditions. This may provide a further explanation of the decrease in conversion observed at higher input HPA concentrations (Fig. 3C). The reactions were each sampled at 2.5 residence times, at between 5 and 12 h (depending on the input HPA concentration); Fig. 4 suggests that the TK activity may have decayed significantly by the time reactions with longer residence times were sampled.

A further modification that could be made to overcome this problem is the addition of a cooling system to maintain the stability of the solubilised enzyme. The enzyme could be thermo-stated to the desired temperature shortly before it is introduced into the reactor. A mutant of the TK enzyme with greater temperature stability could also be used. If the initial concentration of ERY observed in Fig. 4 could be maintained, this would allow production at a rate of 19 mg h^−1^, and thus would have led to a total of 171 mg over the 12 h.

## 4 Conclusion

We have successfully demonstrated the application of ‘fed-batch’ like substrate feeding strategies to continuous-flow microreactor technology to address the limitations of single-point feeding. Our novel flexible microfluidic reactor design offers numerous input configurations and we tested different feeding strategies using the TK-catalysed condensation of HPA and GA as the test reaction. Furthermore, the design is easily fabricated with laser ablation, with the potential for modification to develop customised reactions for different biocatalysts and feeding strategies. The modularity of the design would also allow the incorporation of the MIR into a wider network of reactors and separation technologies, with the potential to develop multi-step synthetic processes for complex molecular products.

Using our substrate feeding strategies and the flexibility of the MIR, we have greatly improved on the yield of product achieved with our previous microfluidic reactor [31]. The output concentration of ERY has been increased from 50 mM to 224 mM ERY, the equivalent of 313 mM if the dilution by GA feeding is compensated for, without exposing the enzyme to destructive concentrations of substrate. In addition, we have shown a 5-fold increase in the throughput of the MIR over our previous reactor, to a maximum of 15 mg h^−1^, with a maximum 8-fold improvement over fed-batch microplate reactions. This highlights the primary advantage of continuous-flow reactors in terms of synthetic productivity.

We have shown that the reactor is capable of very high conversion for short periods of time, but in order to achieve and maintain full conversion for higher input concentrations further improvements are necessary, addressing issues of CO_2_ evolution and enzyme stability. This would allow the synthesis of ERY at a rate which would represent a significant step towards the high throughput and output concentrations that are commonly associated with chemical microreactors.

## Figures and Tables

**Figure:**
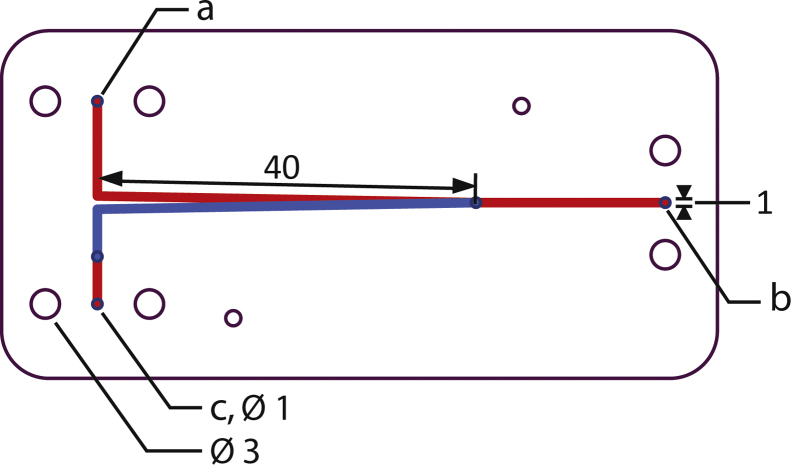


**Figure:**
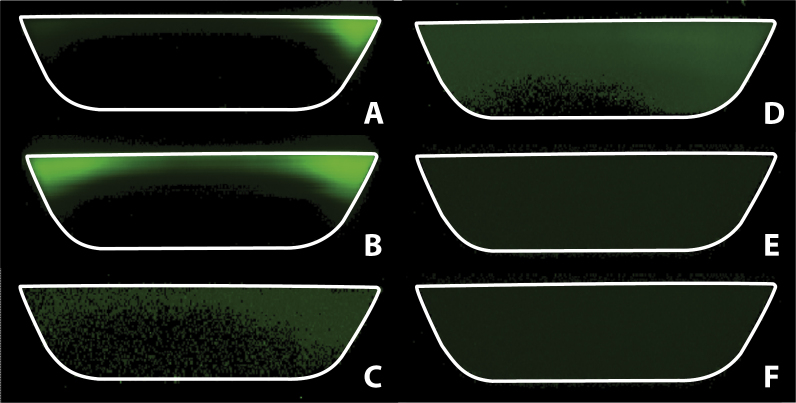


**Figure:**
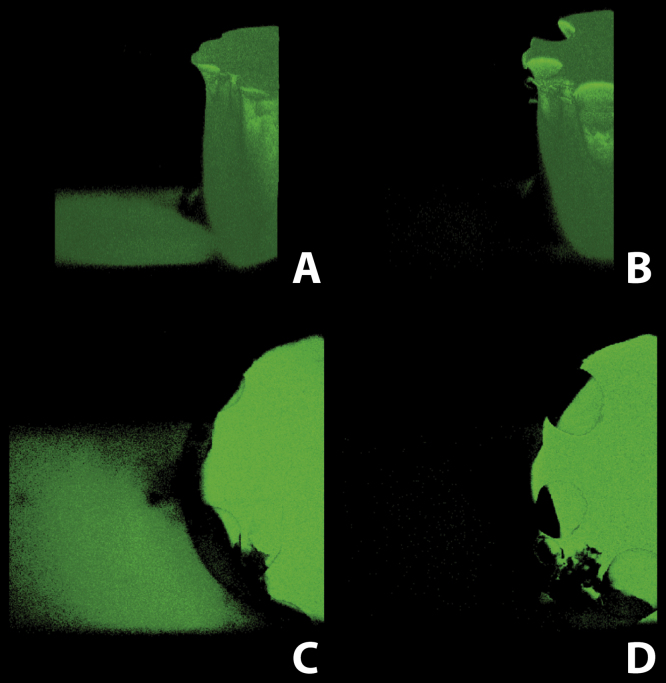


**Table 1:** Reactor configuration. Configuration of reactor inputs, residence times and flow rates used to perform reactions with different input HPA concentrations and residence times.

		HPA input concentration (mM)			
		200	300	400	500
Residence time (mins)		120	165	264	286
Position	Primary 1	TK/HPA	TK/HPA	TK/HPA	TK/HPA
	Primary 2	GA	GA	GA	GA
	Aux 1	GA	GA	GA	GA
	Aux 2	-	-	GA	GA
	Aux 3	-	-	GA	GA
	Aux 4	GA	GA	GA	GA
	Aux 5	-	GA	GA	GA
	Aux 6	GA	-	GA	GA
	Aux 7	-	-	-	GA
	Aux 8	-	GA	-	GA
	Aux 9	-	-	GA	-
	Aux 10	-	-	-	-

Flow rate (μL min^−1^)	Primary 1	4.49	4.94	8.24	11.90
	Primary 2 & Aux	0.24	0.26	0.43	0.63
