# Supplementary material for: Microfluidic multi-input reactor for biocatalytic synthesis using transketolase
Source: J Mol Catal B Enzym. 2013 Nov;95:111–7. doi: 10.1016/j.molcatb.2013.05.016 (PMC3724052; doi:10.1016/j.molcatb.2013.05.016)
Supplement: Supplementary file 1 [file mmc1.pdf]

ESI 2: Confocal imaging

Confocal imaging was used to analyse the rate of diffusion of substrate fed at the auxiliary inputs into the main reaction mixture. A microfluidic chip was designed specifically for this purpose, mimicking the structure of a single auxiliary input and channel section in the MIR, with some modification to accommodate confocal imaging (figure S1). The chip was fabricated primarily in PMMA except for the top plate, which was an adhesive-free microwell plate sealing film, used to facilitate the transmission of light from the interior of the channel.

Water and 0.1 mg ml^{-1}fluorescein in water were flowed through the primary and auxiliaryinputs respectively (labelled a and b in figure S1). Confocal microscopy images were taken 1 and 2 cm downstream from the channel joinwith a Leica TCS SP2 confocal microscope (Leica Microsystems UK Ltd, Milton Keynes, UK). A 10× objective was used with a 488 nm argon laser at 30% power and a FITC filter.The channel was imaged from the top to the floor in horizontal sections 5 microns apart. The flow rates and location of the imaging were adjusted to visualise the diffusion at different residence times, between 1.4 and 60 seconds. The ratio of the water to fluorescein solution flow rates were maintained at 20:1, regardless of the residence time investigated. The sections were compiled into composite images using with Leica Confocal Imaging software (Leica Microsystems UK Ltd) to create vertical cross-sections of the channel.

The confocal images show that the solution fed into the auxiliary input is initially deflected to the outer edges of the reaction channel by the faster-flowing fluid from the primary input (figures S2A and S2B). However, the auxiliary solution begins to diffuse into the bulk fluid quite rapidly, and the two solutions appear to be homogenous within 45 seconds of being combined at the channel join (figures S2C to S2F). In the MIR, this residence time corresponds to less than a single section of the main reactor channel, even at the highest flow rates used in the ERY synthesis experiments.

The images appear to show a relatively smooth channel floor. This has been confirmed by profilometry (mean roughness Rz = 6.8μm) and is in agreement with previously-published work describing the roughness of channels fabricated in PMMA by laser ablation [Klank H, Kutter JP, Geschke O, Lab Chip, 2 (2002)242-6].

The rate of diffusion along the auxiliary channel itself was also evaluated in order to ensure there was no backflow of reaction mixture out of the auxiliary ports. In this case the fluorescein solution was flowed through the primary input (a) and water was flowed into the auxiliary input (b). The join between the channels was imaged across both layers in horizontal sections 10 microns apart, using the same settings as above. Fluorescein solution and water were flowed into the chip in a 20:1 ratio, at 3.3 µl min^{-1} and 0.17 µl min^{-1} respectively. A control image was taken with static fluorescein solution in the main channel and the auxiliary input.

The control images (figures S3A and S3C) clearly show the structure of the channel join, with a column of fluid visible on the right side of the images linking the channels on the top and base layers. The auxiliary input channel is shown on the left side of the images. Under flow conditions (figures S3B and S3D), no fluorescein is visible in the input channel, suggesting that there is no backflow from the main channel. Given that this is around half of the minimum flow rate used in the synthesis experiments, we are confident that this is representative of the flow regime generally.

Figure S1: Design of reactor mimicking single auxiliary input of MIR

Achip to designed for mimic the auxiliary input of the MIR for the purposes of confocal imaging of diffusion after the channel join, showing the location of the primary input (a), auxiliary input (b) and outlet (c). Features in red are on the base layer of the chip, blue are on the middle layer and purple are on both layers. Filled areas indicate channels (1 mm x 0.5 mm), outlines indicate cuts through the substrate. The chip was closed with an upper layer of microplate sealing film. All units are in mm.

Figure S2: Vertical sections of channel showing mixing at various residence times

Confocal imaging was performed at different distances from the channel join and with different flow rates, in order to evaluate the diffusion at several residence times. The residence times evaluated were (A) 1.4; (B) 2.8; (C) 14.8; (D) 29.6; (E) 45.0 and (F) 60.0 seconds. Images were compiled to create vertical cross-sections of the channel. White outlines indicate the channel boundaries; green indicates the spread of fluorescein through the channel.

Figure S3: Imaging of channel join showing absence of backflow

Confocal imaging was performed at the channel join in order to evaluate the whether any backflow of material from the main channel might be occurring. Images were taken with static fluorescein (A and C) and with fluorescein and water flowing through the primary and auxiliary inputs respectively (B and D). Images were compiled to create views from the front (A and B) and the top (C and D) of the join. Green indicates the spread of fluorescein through the channels.
