# Supplementary material for: Microfluidic multi-input reactor for biocatalytic synthesis using transketolase
Source: J Mol Catal B Enzym. 2013 Nov;95:111–7. doi: 10.1016/j.molcatb.2013.05.016 (PMC3724052; doi:10.1016/j.molcatb.2013.05.016)
Supplement: Supplementary file 2 [file mmc2.pdf]

ESI 3: Reaction setup calculation

Michaelis-Mentenkinetics were used to estimate when GA would need to be fed into the reaction mixture in order to complete the conversion of HPA to ERY without exposing TK to GA concentrations higher than 50 mM. A previously-published equation describing the kinetics of the TK-catalysed condensation of HPA and GA was used, along with reported kinetic and inhibitory constants [M. Gyamerah, A.J. Willetts, Enzyme Microb. Tech. 20 (1997) 127-134].

The equation was used in a step-wise fashion to make a crude estimation of the reaction's progress in order to estimate where GA feeding points would be required. The reactor was effectively treated as a series of discrete sections of constant volume, and the reaction rate and substrate and product concentrations were calculated for each section in sequence. In each section the reaction rate was calculated using the output concentrations of the previous section (or the initial conditions if the section in question was the first); the rate was assumed to remain constant over the length of the section. The change in substrate and product concentrations were then calculated and used to determine the reaction rate for the next section.

The lower limit on GA concentration was set at 5 mM. Whenever the concentration was estimated to drop below this limit in a particular section, the feeding of additional GA was factored into the calculation. For the feeding of GA, the auxiliary flow rates were set at 5% of the primary (reaction mixture input) flow rate, allowing the dilution of substrate, product and enzyme to be calculated. It was also assumed that the GA and reaction mixture streams would combine instantaneously, and that diffusion would have a negligible effect on the reaction rate.The auxiliary flow rates were set at 5% of the primary flow rates in order to minimise the dilution of the reaction mixture whilst keeping the flow rates at a level that could be reliably delivered by a syringe drive.

By simulating the feeding of GA every time the concentration dropped below the 5 mM limit, it was possible to estimate the number and timing of GA inputs that would be required to achieve complete conversion to ERY. For each input concentration of HPA to be tested, a mean residence time (of the reaction mixture) was established based upon the estimated time required to achieve a minimum of 97.5% theoretical conversion. The calculated GA feeding time points were then assigned to physical input positions on the reactor, chosen to be closest to the required points estimated. The calculation of end point concentrations overestimated the final concentration of ERY by 15% - 35% for the 200 – 500 mM input HPA concentrations respectively.
