# Supplementary figures and images for: Microfluidic multi-input reactor for biocatalytic synthesis using transketolase
Source: J Mol Catal B Enzym. 2013 Nov;95:111–7. doi: 10.1016/j.molcatb.2013.05.016 (PMC3724052; doi:10.1016/j.molcatb.2013.05.016)

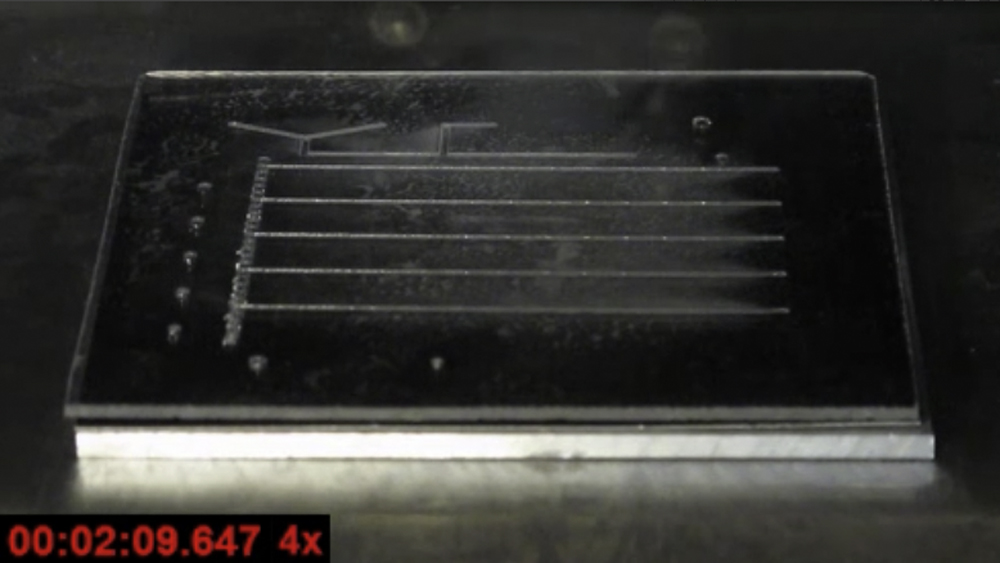

Supplement: Supplementary file 3 [file mmc6.jpg]
